# Feasibility of a quality improvement project to increase adherence to evidence-based pulmonary embolism diagnosis in the emergency department

**DOI:** 10.1186/s40814-020-00741-8

**Published:** 2021-01-04

**Authors:** Federico Germini, Yang Hu, Sarah Afzal, Fayad Al-haimus, Srikanth A. Puttagunta, Saghar Niaz, Teresa Chan, Natasha Clayton, Shawn Mondoux, Lehana Thabane, Kerstin de Wit

**Affiliations:** 1 Department of Health Research Methods, Evidence, and Impact, Health Information Research Unit (HIRU), Communication Research Laboratory (CRL), McMaster University, 1280 Main Street West, Hamilton, ON L8S 4K1 Canada; 2 Department of Medicine, McMaster University, Hamilton, ON Canada; 3 Department of Radiology, McMaster University, Hamilton, ON Canada; 4 Department of Emergency Medicine, St Joseph’s Healthcare, Hamilton, ON Canada; 5 Population Health Research Institute, Hamilton Health Sciences, Hamilton, ON Canada; 6 Biostatistics Unit, Father Sean O’Sullivan Research Centre, St Joseph’s Healthcare, Hamilton, ON Canada; 7 Departments of Paediatrics and Anaesthesia, McMaster University, Hamilton, ON Canada; 8 Centre for Evaluation of Medicine, St Joseph’s Healthcare, Hamilton, ON Canada

## Abstract

**Background:**

Many evidence-based clinical decision tools are available for the diagnosis of pulmonary embolism (PE). However, these clinical decision tools have had suboptimal uptake in the everyday clinical practice in emergency departments (EDs), despite numerous implementation efforts. We aimed to test the feasibility of a multi-faceted intervention to implement an evidence-based PE diagnosis protocol.

**Methods:**

We conducted an interrupted time series study in three EDs in Ontario, Canada. We enrolled consecutive adult patients accessing the ED with suspected PE from January 1, 2018, to February 28, 2020. Components of the intervention were as follows: clinical leadership endorsement, a new pathway for PE testing, physician education, personalized confidential physician feedback, and collection of patient outcome information. The intervention was implemented in November 2019. We identified six criteria for defining the feasibility outcome: successful implementation of the intervention in at least two of the three sites, capturing data on ≥ 80% of all CTPAs ordered in the EDs, timely access to electronic data, rapid manual data extraction with feedback preparation before the end of the month ≥ 80% of the time, and time required for manual data extraction and feedback preparation ≤ 2 days per week in total.

**Results:**

The intervention was successfully implemented in two out of three sites. A total of 5094 and 899 patients were tested for PE in the period before and after the intervention, respectively. We captured data from 90% of CTPAs ordered in the EDs, and we accessed the required electronic data. The manual data extraction and individual emergency physician audit and feedback were consistently finalized before the end of each month. The time required for manual data extraction and feedback preparation was ≤ 2 days per week (14 h).

**Conclusions:**

We proved the feasibility of implementing an evidence-based PE diagnosis protocol in two EDs. We were not successful implementing the protocol in the third ED.

**Registration:**

The study was not registered.

## Key messages regarding feasibility

- We were not sure data could be accessed in a timely manner, and to what extent they could be automatically downloaded from the hospital’s database. Moreover, we were not sure if our inclusion criteria were capturing the right population.
- One of the three participating centers could not implement the intervention, mainly for lack of dedicated financial and human resources. Substantial efforts were required to access the electronic data, and some of the data had to be extracted manually.
- The main changes implemented after the feasibility assessment were as follows: adapting the inclusion criteria, reducing the frequency of the feedback, and making the feedback content lighter and simpler to understand.

## Introduction

### Background

The diagnosis of pulmonary embolism (PE) is a multi-step process. At least 10 different clinical decision tools are available [1], mainly aimed at reducing the use of advanced imaging techniques such as computed tomography pulmonary angiography (CTPA) or ventilation-perfusion scan (VQ). When PE prevalence is 15%, clinician’s Gestalt, the Wells score, and the Revised Geneva score all have an estimated sensitivity greater than 80%. When combined with D-dimer testing, the failure rate for PE diagnosis is below 2% [1]. These standards are now used to benchmark the safety of new PE tests. Clinical decision tool use is endorsed by recent guidelines [2]. Despite high-quality evidence supporting the use of these diagnostic algorithms, published data show that these tools are seldom used in everyday clinical practice [3–6]. The main effect of this is the overuse of CTPA, which translates into excess radiation exposure, the possibility of contrast-induced nephropathy, overtreatment, and reduction of health system efficiency and resources. According to some simulations, 0.6 to 2.0% of all cancers in the USA may be attributable to the radiation from CT studies [7, 8]. Contrast-induced nephropathy is reported in 1–2% of patients with normal renal function and 5% of patients with chronic renal failure undergoing an intravenous low-osmolality contrast-medium injection for computed tomography [9]. Contrast-induced nephropathy is associated with increased mortality, need for dialysis, and longer hospital stays [10]. The knowledge translation gap persists despite numerous efforts to improve the implementation of evidence-based diagnostic pathways [6, 11]. The reasons why implementation studies to date had minimal or no impact on the use of CTPA include the perception that clinical decision support systems are complicated, have a negative impact on productivity, are not supported by a sufficient body of evidence, and are not better than clinical judgment [6, 11]. Moreover, defensive behaviors, such as “fear of missing PE,” have been identified as associated with a lower CTPA positive yield [12], and might be a barrier to the implementation of quality improvement interventions. This tendency toward overtesting seems to be more rooted in the North American environment, than in European [13].

#### Why the evidence-practice gap?

de Wit and colleagues conducted a nationwide think-aloud interview study with 63 emergency department (ED) physicians from nine sites (unpublished data). This study found that the sources of variance in decision-making arose from the following: (1) physicians’ risk tolerance for missing a PE diagnosis being very low, (2) physicians are confident in their gestalt and think their suspicion is sufficient to order a CTPA, (3) the Wells score is perceived as complicated and can lead to either under- or overestimation of clinical probability (this might apply to the other tools, but only the Wells score was investigated in this study), and (4) having to order a D-dimer blood test, waiting for the results, and then ordering a CTPA if needed is perceived as delaying an inevitable scan. Using the Theoretical Domains Framework, this same group interviewed ED physicians within 2 weeks of them having deviated for evidence-based protocol for PE diagnosis (unpublished data). They found the following themes: (1) reserved confidence in the clinical guidelines and ability to apply them, (2) belief that strong clinical gestalt is crucial when testing for PE, (3) fear of not diagnosing PE, and (4) belief that the advantages of the standard protocol outweigh the disadvantages. Lastly, our research group has also performed semi-structured interviews with ED patients who were being tested for PE [14]. Patient interviews revealed zero tolerance for false positive and false negative diagnoses, association of rapid ED assessment with better quality testing, preference for individualized testing, contradictory acceptance of CT limitations for PE diagnosis, overestimation of pretest probability for PE, association between more tests with better quality testing, preference for imaging over clinical examination to exclude PE, primary concern being testing related to previous heart-related issue, a focus on pain symptoms rather than underlying diagnosis, and a preference for direct interaction with ED physician.

It is time to create a new implementation method for evidence-based PE diagnosis in the ED. An effective strategy would be safe for patients, use resources judiciously, and benefit physicians as well as patients. Considering the barriers to the successful implementation of evidence-based diagnostic strategies highlighted in previous studies [6, 11, 14], we thought it is crucial to use a clinical decision tool that is simple, safe, and supported by high-level quality of evidence. Furthermore, we decided to design a multi-faceted intervention, ensuring leadership endorsement and targeting patients and healthcare workers, with a focus on physicians. However, given the challenges and the negative results from previous quality improvement studies for PE diagnosis, we decided to assess the feasibility of our intervention in two centers in Hamilton, ON, and one in Ottawa, ON, before attempting to assess its effect or to implement the protocol on a larger scale. This report will focus on the feasibility aspects of the study.

The primary objective of this study was to assess the feasibility of implementing an evidence-based PE diagnosis protocol in three EDs. The secondary objective was to report data on the period preceding the intervention and preliminary data on the first 3 months post-intervention.

## Methods

### Study design

This is a study on the feasibility of a quality improvement intervention, with a before-after design.

### Context/study setting

The study was conducted in three EDs: Hamilton Health Sciences (HHS) EDs (Hamilton General and Juravinski Hospitals, Hamilton, ON) and the Montfort Hospital, Ottawa, ON. These are teaching hospitals and are staffed with approximately 50 physicians (Hamilton Hospitals) and 32 physicians (Montfort Hospital) who manage 100,000 and 55,000 patient visits per year, respectively. A chart review of HHS EDs 2013–2015 showed that 290 patients were investigated for PE in the HHS EDs every 6 months [15].

### Population studied

This implementation study used electronic data to identify patients tested for PE in the ED. The population was consecutive adults (aged 18 years and older) with suspected PE, for whom a D-dimer blood test and/or imaging for PE (CTPA or VQ scan) were performed in the ED. When a patient books into the ED, the triage nurse assigns a chief complaint from a selection of predefined categories, classified based on the Canadian Emergency Department Information Systems (CEDIS) Presenting Complaint List [16]. D-dimer blood test is used to diagnose both deep vein thrombosis and PE, so to ensure we captured only patients tested for PE (and not deep vein thrombosis), we aimed to restrict our population to those who presented to the ED with the presenting complaint “chest pain” (cardiac and non-cardiac) and/or “shortness of breath.” To evaluate whether a sufficient proportion of all patients tested for PE were registered under these two presenting complaints, we retrieved a list of CTPAs ordered in the ED in 2018. We manually extracted the presenting complaints for each case. We aimed to capture a minimum of 80% of the CTPAs ordered in the ED. The rationale for the use of predefined triage categories to identify the study population was to reduce inter-person variability in this process.

### Implementation

We led a Canadian Association of Emergency Physician (CAEP) working group consisting of six emergency physicians from across Canada with expertise in PE diagnosis and knowledge translation. This knowledge broker group systematically reviewed the literature and identified all optimal PE diagnostic strategies for the ED, as well as optimal ways to encourage adherence to this diagnostic strategy. As a result, we decided to test a multimodal intervention aimed at promoting the uptake of D-dimer in everyday clinical practice. The implementation strategy is based on the knowledge translation recommendations from CAEP [17]. The components of the intervention are detailed in Table 1. This implementation strategy was discussed at each site by engaging with local champions, hospital managers, nurses, diagnostic imaging staff, support staff, and physicians to identify and implement strategies to overcome local barriers.

**Table 1:** Description of the components of the intervention

**Leadership endorsement** We obtained approval from the clinical and managerial leads for the ED, radiology, hematology, and thrombosis for a new protocol for the diagnosis of PE.	
**Ordering D-dimer and CTPA/VQ scan** We moved from the concept of ordering D-dimer or imaging for PE, to the broader concept of “testing for PE.” We created a new order set (Appendix A) which guides ED testing for PE. The new diagnostic PE pathway starts with D-dimer blood testing in all patients. We no longer asked the physician to calculate the Wells score to simplify the process and to avoid having physicians artificially increasing the score in order to avoid using D-dimer. The testing process has been semi-automated. If the D-dimer result is lower than the threshold, the attending physician is notified by the nurse and PE is excluded. If the D-dimer result is higher than the threshold, the patients go directly for a CTPA without the need for physician reassessment. The physician is notified when the imaging report is available. We made the new PE diagnostic pathway attractive to use by enabling ordering of CTPA without the requirement to first discuss with a radiologist.	
**Physicians’ education** We met with the ED physicians and nurses with educational material to support the use of the proposed diagnostic workflow.	
**Personalized confidential physician feedback** We sent each physician a quarterly confidential personalized report containing the following: The proportion of eligible patients (based on the presenting complain) who had an imaging test, expressed as a percentage: (number of exams requested) × 100/(total number of eligible patients). The proportion of imaging tests ordered without D-dimer or despite a negative D-dimer, expressed as a percentage: (number of cases in which the algorithm has not been followed in patients receiving imaging) × 100/(total number of imaging test performed). These metrics were calculated for the individual physician, and compared to the average of all the physicians working in the same ED. The form was piloted with some of the study clinical investigators (the research manager and two ED physicians with expertise in quality improvement and knowledge translation) and then with a convenience sample of four physicians. The form was modified according to their feedback.	
**Patients’ information** We developed patient information about the testing process, as well as the risks and benefits of undergoing CT scanning. Moreover, the PE testing order set incorporated nurse facilitated identification of patient-specific goals (for example treatment of pain) so the treating ED physician can focus their treatment and advice on patient-specific needs.	

*ED* emergency department, *PE* pulmonary embolism, *CTPA* computed tomography pulmonary angiography

### Comparison and timelines

**Fig. 1:**
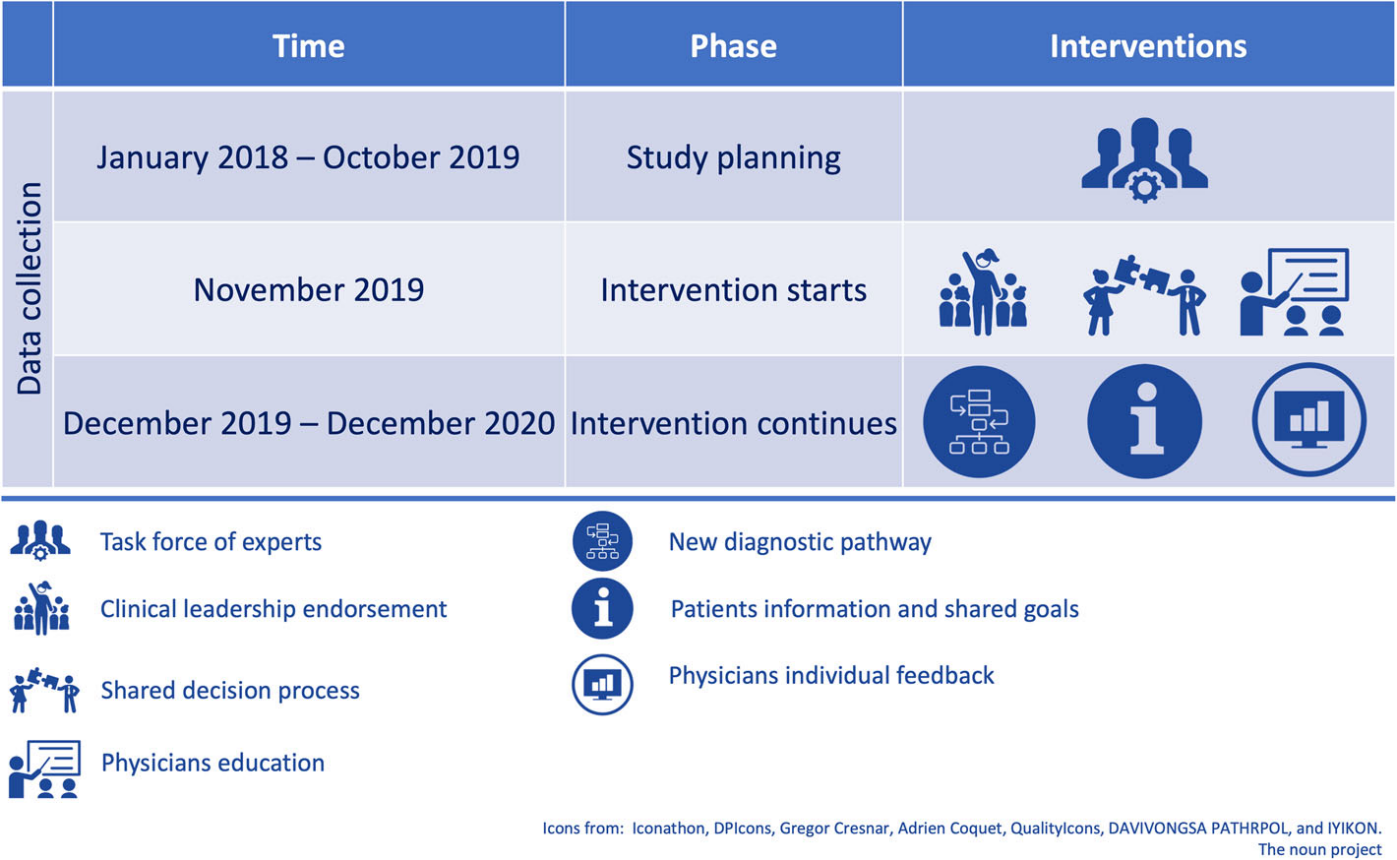
Flow chart describing the components of the intervention

A flow chart describing the timeline is reported in Fig. 1. Data on baseline clinical practice were collected from January 1, 2018, to October 31, 2019. The intervention was implemented in November 2019. Data on the period after the comparison were collected starting in December 2019, since November was considered as a run-in period (this was specified before accessing the data). For the purpose of this report, we present the post-implementation data up till the end of February 2020.

### Outcomes

#### Primary outcome—feasibility

Feasibility was described using 6 criteria, established through discussion between the study investigator:

1. Implementation of the new diagnostic PE protocol at the participating hospitals (yes/no for each center). To consider the intervention feasible, it should have been implemented in at least two of the three participating sites.
2. Electronic identification of our population of interest capturing ≥ 80% of all CTPAs ordered in the EDs.
3. Establishing access to the required electronic data with monthly data updates (yes/no).
4. Timely manual chart data extraction. To be considered feasible, the data extraction had to be completed 5 days before the end of the following month ≥ 80% of the times.
5. Implementation of individual emergency physician audit and feedback. To be considered feasible, we required that feedback data on the previous month was complete before the end of the following month ≥ 80% of the time.
6. An estimate of the number of hours of research assistant time to extract the required data and synthesize the physician feedback reports (total number of hours per week). To be considered feasible if ≤ 2 days/week.

#### Secondary outcomes—preliminary estimates of effect

The outcomes used for the preliminary estimate of the effect of the intervention were the following:

a Proportion of patients tested for PE among the whole study population.
b Proportion of patients tested for PE in adherence to the protocol among the total number of patients tested for PE.
c Proportion of eligible patients with an imaging test.
d Diagnostic yield of imaging tests requested: (number of exams positive for PE) × 100/(total number of exams requested).
e Proportion of imaging tests ordered without D-dimer or despite a negative D-dimer: (number of cases in which the algorithm was not followed in patients receiving imaging)/(total number of imaging test performed).
f Proportion of imaging tests not ordered, despite D-dimer positivity: (number of cases in which imaging was indicated and not performed)/(total cases in which imaging was indicated).
g We also described the prevalence of PE, as follows: all PEs, central PE (segmental or more), and distal PE (sub-segmental).

#### Balancing measure

This is a before-after comparison of the number of D-dimer blood tests ordered in the ED.

### Analysis

Baseline patient characteristics and the feasibility measures were reported using standard descriptors of central tendency and variability (mean and standard deviation or median and ranges as appropriate). The secondary outcomes regarding effect and balancing measure were reported descriptively, with the 95% confidence intervals (CIs) for the proportions’ differences. To facilitate visual inspection, the outcomes were also plotted against time with two regression lines, before and after the intervention. All the analyses were conducted with STATA/IC v. 16 (StataCorp LP, College Station, TX, USA). *We intentionally avoided formally testing our hypothesis, because the study is continuing.*

### Ethics

Research ethics approval was obtained from participating sites prior to commencing the study (Hamilton Integrated Research Ethics Board # 5339-C).

## Results

### Primary outcome: feasibility of the study

A summary of the results for the feasibility of the study is reported in Table 2.

**Table 2:** Results for the feasibility of the study

Outcome	Criteria for feasibility	Feasibility proved
Implementation of the new diagnostic PE protocol at the participating hospitals	Successful implementation in at least two of the three participating sites	Yes, intervention implemented in two centers
Identification of the population	Capturing ≥ 80% of all CTPAs ordered	Yes, 90% captured
Access to all the required electronic data	Successful access to all the data	Yes
Timely manual data extraction	Completed 5 days before the end of the following month ≥ 80% of the times	Yes, 100% of the times
Implementation of individual emergency physician audit and feedback	Feedback on the previous month ready to be emailed before the end of the following month ≥ 80% of the times	Yes, 100% of the times
Number of hours of research assistant time to extract the required data and synthesize the physician feedback reports	≤ 2 days/week	Yes, 14 h/week

*PE* pulmonary embolism, *CTPA* computed tomography pulmonary angiography

#### Implementation at participating hospitals

**Fig. 2:**
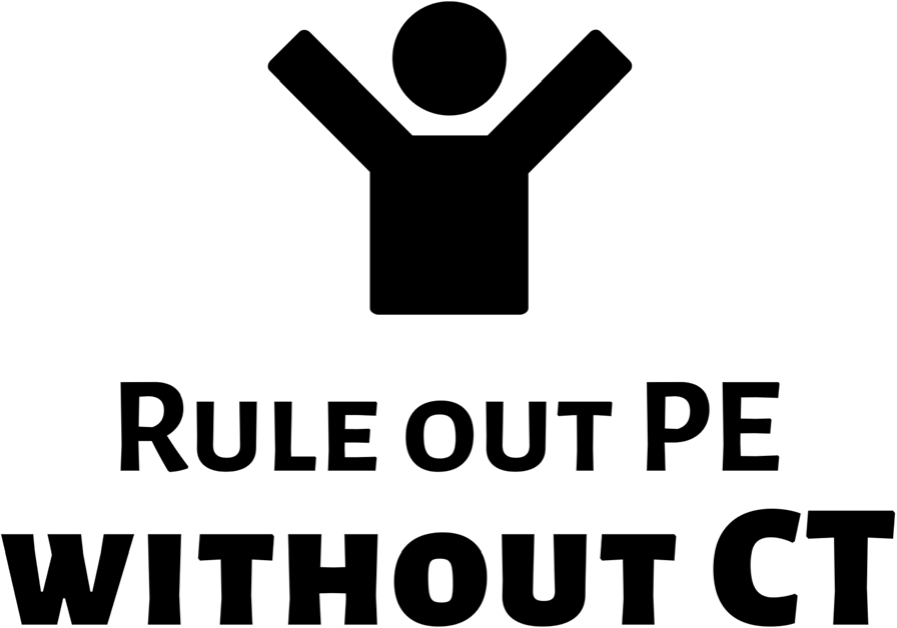
Sticker attached to the computers in the ED physicians’ office

The intervention was implemented at the Hamilton sites, but not at the Montfort Hospital in Ottawa. The PE diagnostic order set was initiated on October 28, 2019. The topic of PE diagnosis was discussed at the HHS ED physicians’ rounds, and three educational podcasts were recorded and remain freely available for streaming and download [18–20]. All emergency physicians received an email explaining the rationale for the intervention and its objective, accompanied by a Frequently Asked Question section and educational material (email text available in Appendix B). As a reminder to the ED physician group, we attached laminated stickers with a logo and the invitation to “rule out PE without CT” to each computer in their offices (Fig. 2). A team of three nurse educators engaged in individual meetings with the ED nurses, explaining the aim of the intervention and the new workflow. A multidisciplinary team including managers, radiologists, emergency physicians, educators, radiation technologists, and nurses met monthly to review progress and problems arising with the new order set. A number of logistical changes were made including automatic population of the CTPA request form with the D-dimer result and estimated glomerular filtration rate for the radiology technicians and streamlining of process.

We collaborated with regular project meetings with the Montfort Hospital ED. In May 2019, our colleagues let us know they were not able to participate in the study due to lack of data access and resources.

#### Identification of the population

We found that the two selected CEDIS presenting complaints (chest pain and shortness of breath) only captured 70% of all CTPAs ordered by emergency physicians. Therefore, inclusion criteria were expanded to 13 presenting ED presenting complaints (Table 3), allowing us to capture 90% of the ED-ordered CTPAs. The remaining 10% were dispersed among 40 presenting complaints, each one accounting for 0.1–0.9% of all CTPAs (Table S1).

**Table 3:** Distribution of CTPAs by presenting complaint in 2018

CEDIS description	CTPAs (*n*)	CTPAs (%)
Chest pain (cardiac features)	261	25
Chest pain (non-cardiac features)	98	9
Shortness of breath	366	35
Palpitations/irregular heart beat	43	4
Syncope/pre-syncope	30	3
Hemoptysis	16	2
Cardiac arrest (non-traumatic)	7	1
Respiratory arrest	1	0
General weakness	43	4
Back pain	24	2
Cough/congestion	18	2
Abdominal pain unspecified	25	2
Hyperventilation*	0	0
Other	105	10
Total	1037	

*CEDIS* Canadian Emergency Department Information System, *CTPA* computed tomography pulmonary angiography

*Hyperventilation was added even if it is seldom used in our emergency departments and no CTPA was ordered. The rationale is that it can be a typical presentation of PE

#### Obtaining electronic data

We requested data from three sources: the hospital decision support services, an internal hospital research database, and the eHealth Information Technology Services (eHITS) office. The first two sources were unable to provide timely data (at the end of each month). The eHITS department was able to provide us with the required data in the required turnaround time. After working together to define the database queries and to validate the data, we received the first finalized dataset in January 2019. The system is now automated with monthly updates.

#### Manual data extraction

We found the variable “ordering doctor” for the CTPAs in the electronic medical record (EMR) was not accurate due to some scans being ordered by an admitting service but the order was logged under the emergency physician’s name. It was crucial for us to have accurate data for the ordering physician, or the physician audit and feedback would lose credibility. Therefore, we manually checked the ordering physician data. Despite this increase in the workload, the manual extraction was always completed before the end of the following month.

#### Implementation of individual physician feedback

**Fig. 3:**
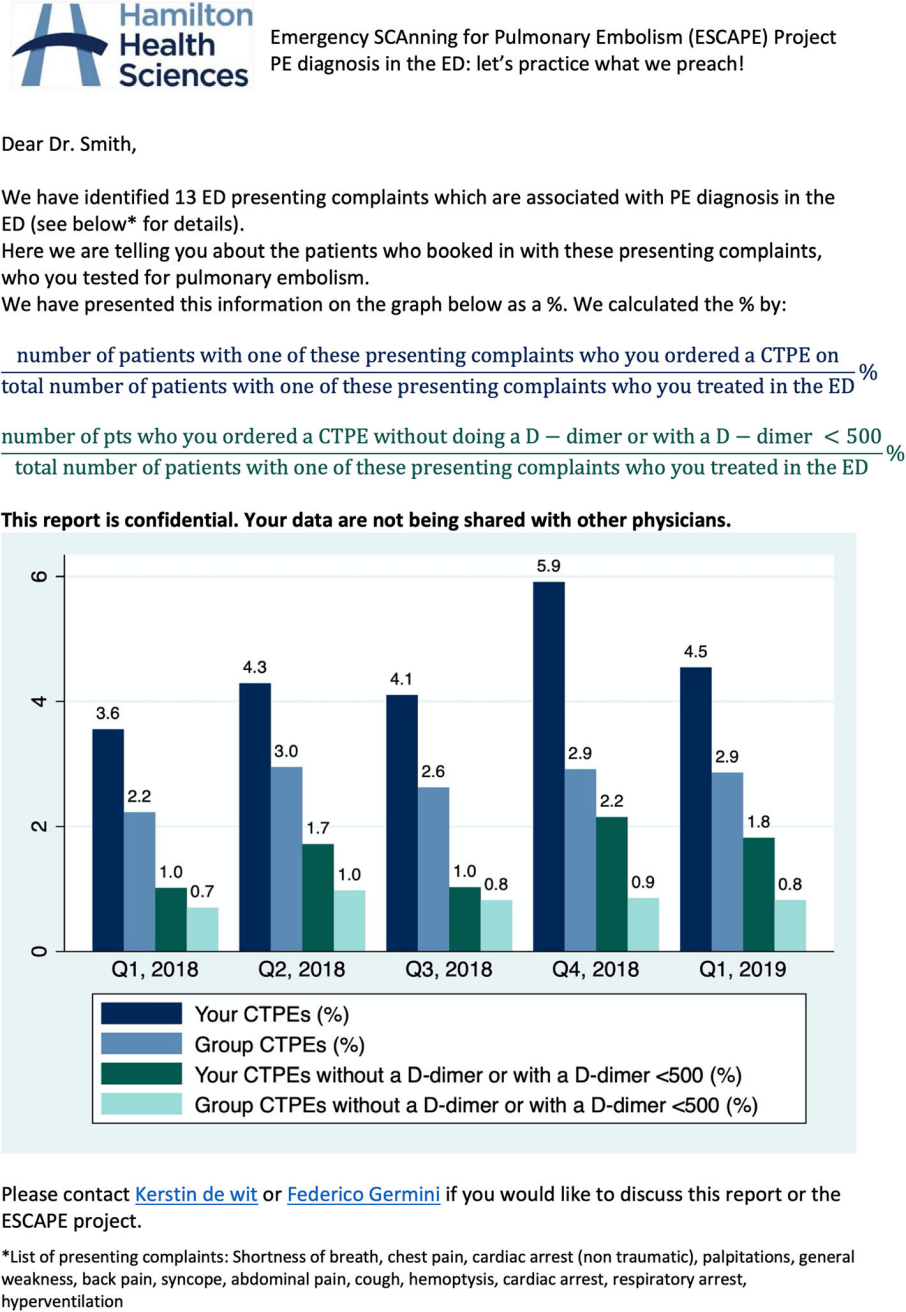
An example of the personalized physician feedback

Audit and feedback intervention are effective for improving healthcare professionals’ compliance with desired practice [21], and individual feedback has a potential additional role as compared to group feedback alone [22]. The path toward the implementation of physician feedback proved to be challenging, and many adaptations of the original plan were required. Initially, we aimed to provide individual feedback to physicians on a monthly base. When reviewed, we realized that the number of CTPAs ordered per month per physician was too low (range 0–6 CTPAs per month per physician). The proportion of inappropriate CTPAs would have been subject to enormous variability for very small variations in the actual number of non-appropriate CTPAs ordered. We decided to reduce the frequency of feedback from monthly to quarterly, and the feedback was issued at the end of the first 3 months post-implementation. An example of the physician feedback is reported in Fig. 3.

#### Estimate of research assistant time

Based on the first 3 months, we calculated that a research assistant is required for 12 h per week on the project. In addition, a physician should spend 2 h per month to resolve queries. Preparing and checking individual feedback require an additional 6 h per month, on average. Therefore, the total amount of time required to complete these tasks was approximately 14 h per week (less than 2 days), thereby meeting the feasibility criterion.

### Secondary outcomes: preliminary estimates of effect

The patients’ characteristics and outcome distribution are reported in Table 4. In total, 81,103 patients accessed the HHS EDs for one of the selected presenting complaints between January 1, 2018, and February 28, 2020 (70,932 in the before-intervention period and 10,171 after the intervention). Of these, 5993 patients were tested for PE and 2267 patients underwent CTPA or VQ scanning. A total of 285 patients (0.4% of the study population) were diagnosed with acute PE.

**Table 4:** Characteristics of the study population (all patients accessing the ED with a relevant presenting complaint), effect outcome, and balancing measure

	Denominator (for proportions)	Before, ***N*** (%)*	After, ***N*** (%)*	Percentage difference for outcome (95% CI)
**Total no. of patients (*n*)**	NA	70,932	10,171	NA
**No. of patients per month [median (Q1, Q3)]**	NA	3073 (3206, 3324)	3038 (3437, 3696)	NA
**Age, years [median (Q1, Q3)]**	NA	58 (40, 73)	58 (40, 72)	NA
**Female,** ***n*** **(%)**	All patients	38,120 (54%)	5519 (54%)	NA
**Outcomes**				
**Tested for PE**	All patients	5094 (7.2)	899 (8.8)	1.6 (− 0.3; 3.4)
**Test appropriate**	Patients tested for PE	3782 (74.2)	740 (82.3)	8.1 (5.0; 11.2)
**Imaging use**	All patients	1903 (2.7)	364 (3.6)	0.9 (− 1.1; 3.4)
**Imaging positive yield**	Patients with imaging	247 (13.0)	38 (10.4)	− 2.6 (− 13.1; 8.0)
**Imaging not appropriate**	Patients with imaging	432 (22.7)	22 (9.1)	− 13.6 (− 26.3; − 0.9)
**Imaging missed**	Patients with positive D-dimer	665 (35.0)	98 (26.0)	− 9.0 (− 18.4; 0.4)
**PE prevalence**	All patients	247 (0.4)	38 (0.4)	0.0 (− 2.0; 2.1)
**Central PE**	All patients	221 (0.3)	34 (0.3)	0.0 (− 2.0; 2.1)
**Distal PE**	All patients	26 (0.04)	4 (0.04)	0.0 (− 2.1; 2.1
**D-dimer use**	All patients	4643 (6.6)	862 (8.5)	1.9 (− 0.1; 3.9)

*CI* confidence interval, *Q1* first quartile, *Q3* third quartile, *PE* pulmonary embolism

*Unless otherwise specified

**Fig. 4:**
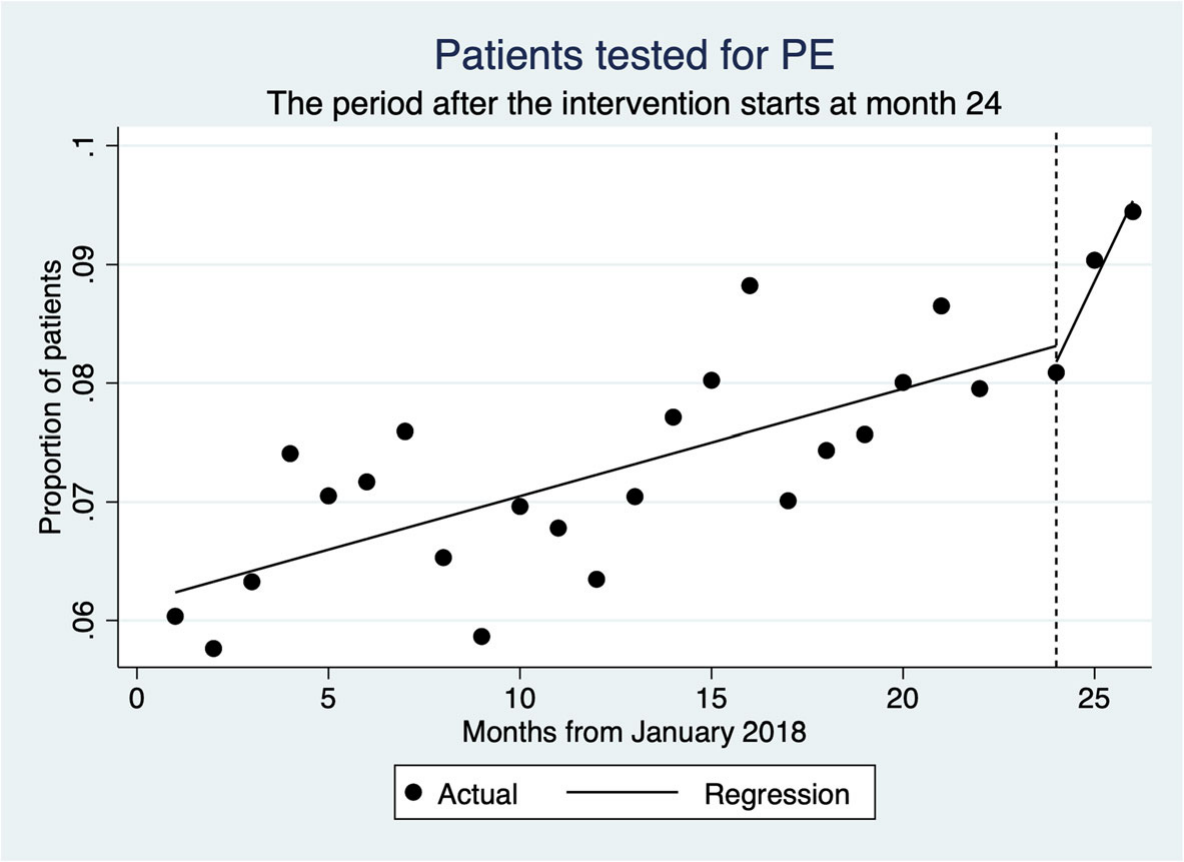
Time trend of testing for PE. The aim of this figure is to display our collected data to show how we will analyze the data in the future. The regression line for the period after the intervention is based on only three points and might not be a reliable estimate of the effect of the intervention

7.2% and 8.8% of the study population were tested for PE before and after the intervention, respectively. There was an 8.1% (95% CI 5.0; 11.2) increase in the adherence to the proposed protocol. The imaging positive yield showed a trend toward reduction (− 2.6%, 95% CI − 13.1; 8.0). The time trends for PE testing are reported in Fig. 4, and the time trends for the remaining secondary outcomes are reported in the appendix (Figures S1, S2, S3, S4, S5 and S6).

### Secondary outcomes: balancing measures

In our study population, a D-dimer was ordered in 6.6% and 8.5% of the patients before and after the intervention, respectively.

## Discussion

In our study, we proved the feasibility of implementing an evidence-based PE diagnosis protocol in the two EDs in Hamilton, ON. The implementation was not successful at a hospital in Ottawa, ON, due to the lack of availability of dedicated resources for data access and manual data extraction. We were able to obtain timely electronic data which identified 90% of the CTPAs ordered in the Hamilton EDs. We found that this implementation protocol takes approximately 14 h per week of research assistant and investigator time. While we successfully implemented the intervention in two out of three centers, we faced numerous barriers. We were expecting to meet some resistance to change due to bureaucracy and human nature, but this translated in delays greater than expected: we were aiming at implementing the intervention in May 2019, but we actually implemented it in November 2019. We encountered measurement barriers, in that it took some time to identify a timely source of electronic data, which required additional manual chart extraction.

The secondary outcome preliminary results showed a signal of improved adherence to the proposed intervention, with fewer imaging tests ordered without D-dimer or despite a negative D-dimer, and more imaging tests ordered after a positive D-dimer. However, for now, this did not translate into a reduction of the use of imaging tests, nor in an increase of the positive yield of imaging. The results suggest an increase in the use of D-dimer and imaging tests, and the prevalence of PE in the population remained the same. It is still too soon to claim that the intervention is futile, but these preliminary results should not be ignored. For this intervention to be meaningful and before scaling it up to a multicenter study, we will need to carefully assess its effect in our population. Historically, most implementation strategies have failed to reduce the number of imaging tests and improve the diagnostic yield of imaging [11]. By embedding a clinical decision support tool for ordering CTPAs in the computerized order entry system, Prevedello et al. showed a small reduction in the use of CTPAs (from 26.5 to 24.3/1000 patient visits, *p* < 0.2) and an increase in the yield of CTPAs (from 9.2 to 12.6%, *p* < .01) [23]. Later on, the same research group failed to further improve these outcomes implementing a performance feedback report for ED physicians. One explanation for our finding may be that we chose not to implement an adjusted D-dimer strategy (such as the YEARS algorithm [24], clinical probability [25], or age-adjusted [26] D-dimer). We chose this plan for simplicity. It may be that implementing an adjusted D-dimer strategy would have been more effective and we are considering changing the intervention in this direction.

### Strengths

Strengths of our study are as follows: (1) the careful review of the existing evidence on the topic and the mixed methods research program that preceded and informed the design of the intervention, (2) the involvement of a multidisciplinary team both for designing and endorsing the intervention, (3) the multi-faceted nature of the intervention aimed at tackling the problem from several angles, (4) the piloting and consequent adaptation of the intervention, and (5) the assessment of its feasibility.

### Limitations

The main limitation of the study is the before-after nature of the comparison. The results might be biased by confounders that changed over time. The generalizability of the study results, both in terms of feasibility and evaluation of the effect of the intervention, may be reduced because the intervention was implemented only in Hamilton EDs. A multicenter step-wedge trial would have mitigated these limitations. For example, the ongoing COVID-19 pandemic appears to be changing the population attending EDs. We expect to see a reduction of visits for complaints that are not related to COVID-19. We also expect the ED personnel to work under a higher level of stress, which might jeopardize the adherence to our protocol. Another limitation is that we were not able to present data on the safety of the intervention. To address this limitation, we are collecting data on the EDs access of any patient with PE in the 30 days preceding the diagnosis. This will increase the data extraction workload but will allow us to find out if these patients could have been diagnosed before and were not, and if this is due to lack of adherence to the protocol or to insufficient safety of the protocol itself.

### Future directions

We will continue implementing and improving the intervention until July 2021, at which point we will have a firm understanding of how to maximize adherence to evidence-based PE testing. *We estimated the cost to run the study in three sites for 1 year to be 55,000 CAD, equating to 0.1 full time equivalent (FTE) of a research coordinator, 0.6 FTE of a research assistant, and 0.04 FTE of a statistician. We recently secured funding by being awarded with the DxQI Seed Grant from the Society to Improve Diagnosis in Medicine (SIDM).*

## Conclusions

We proved the feasibility of implementing an evidence-based PE diagnosis protocol in two Hamilton EDs, but we were unable to implement the protocol in an Ottawa ED. The lessons we learned could be useful to researchers willing to implement similar interventions in the future.

## Supplementary Information

**Additional file 1: Appendix A**. PE testing order set. **Appendix B**. Text of the email sent to the ED physician before introducing the order set. **Table S1**. **Figure S1**. Time trend for testing in adherence to the protocol. **Figure S2**. Time trend for positive yield of image tests. **Figure S3**. Time trend for imaging tests ordered not following the protocol. **Figure S4**. Time trend for PE prevalence. **Figure S5**. Time trend for prevalence of central PE. **Figure S6**. Time trend for prevalence of distal PE.

## Data Availability

The datasets used and/or analyzed during the current study are available from the corresponding author on reasonable request.

## Abbreviations

CEDIS: Canadian Emergency Department Information System
CI: Confidence interval
CTPA: Computed tomography pulmonary angiography
ED: Emergency department
EHR: Electronic health record
HHS: Hamilton Health Sciences
PE: Pulmonary embolism
VTE: Venous thromboembolism
VQ scan: Ventilation/perfusion scan
