# Supplementary material for: Feasibility of a quality improvement project to increase adherence to evidence-based pulmonary embolism diagnosis in the emergency department
Source: Pilot Feasibility Stud. 2021 Jan 4;7:4. doi: 10.1186/s40814-020-00741-8 (PMC7779326; doi:10.1186/s40814-020-00741-8)

Appendix A: PE testing order set

Hamilton Health Sciences

ADDRESSOGRAPH

Patient's Name:

Emergency Department Pulmonary Embolism Testing Order Set

Weight ________ kg

☐ CT Chest with contrast: Query: Pulmonary Embolism (MD to complete requisition **now)**
☐ Consent for contrast attached (if patient unable to give consent)
☐ Provide Testing for PE Information Sheet

Diet: ☐ Regular diet
Activity: ☐ AAT
Vitals: ☐ T, HR, RR, BP, SpO_2 q4h
Lines: ☐ Minimum size 18 gauge PIV or 20 gauge Diffusics IV

IV Fluids

☐ Saline Lock ☐ ____________________

Lab Investigations

☐ ED BASIC
☐ D-DIMER
☐ Urine ß HCG (for female patients between 10 yrs and 55 yrs old)

Diagnostics

☐ Arrange CT if:

- The D-dimer is greater than 500 mcg/L

AND

- The eGFR is greater than 29 mL/min/1.73 m^2

☐ Update status event for physician reassess (RA-MD) if:

- D-dimer is less than 500 mcg/L

OR

- D-dimer is greater than 500 mcg/L AND the eGFR is less than 30 mL/min/1.73 m^2

OR

- CT results are available

*****Complete all areas in signature box. Orders will not be processed without a written signature and bradma on each page*****

Signature: ____________________ Pager # ________ Date ____________ Time ________
Signature/Printed Name/Designation (YYYY/MM/DD)

Co-Signature: ____________________ Pager # ________ Date ____________ Time ________
Signature/Printed Name/Designation (YYYY/MM/DD)

Transcribed By: ____________________ Date ____________ Time ________
Signature/Printed Name/Designation (YYYY/MM/DD)

Checked By: ____________________ Date ____________ Time ________
Signature/Printed Name/Designation (YYYY/MM/DD)

☐ **Copy Made For Pharmacy**

Hamilton Health Sciences

ADDRESSOGRAPH

Patient's Name:________________________

Appendix: YEARS criteria for Pulmonary Embolism

YEARS Criteria (**physician: choose all that apply**)

☐ Hemoptysis present
☐ Leg swelling and tenderness present
☐ PE is the most likely diagnosis
OR
☐ None

This information is being used to evaluate the safety of the YEARS criteria in testing for pulmonary embolism and will not affect current patient care management

Signature: ________________________ Pager # ________ Date ____________ Time ________
Signature/Printed Name/Designation (YYYY/MM/DD)

Co-Signature: ________________________ Pager # ________ Date ____________ Time ________
Signature/Printed Name/Designation (YYYY/MM/DD)

Appendix B

Text of the email sent to the ED physician before introducing the order set:

Hello everyone,

I have been working on improving our testing for pulmonary embolism (PE) for 3 years now, and as of next Monday, 28th October, we will have a new emergency department pathway for testing for PE. I am asking you to change your practice and use this order sheet.

Emergency Department Pulmonary Embolism Testing Order Set_ast_Oct_23_2019_jd.pdf

The major practice change is that every patient tested for PE will first have a D-dimer test.

If the D-dimer is >500 and eGFR >29, the nurse will arrange the CTPE.

The patient will not be flagged for MD reassessment until either the D-dimer is <500 (in which case you do NOT need a CTPE), or the eGFR is <30 (you need to get patient consent for the CTPE), or the CTPE report is ready.

There will be bundles of PE testing orders in the physician office and in RAZ at HGH and the Juravinski.

I do not intend to put these in UCC since patient processing for CT is different there.

Each bundle contains:

1. The order set (link above and also attached)
2. A CTPE requisition which you sign at the start. It will not be used if the D-dimer is <500. This allows the nurse to arrange the CTPE without you having to speak to a radiologist.
3. The YEARS questions - we will analyze whether we can safely implement YEARS at a later date using this information
4. A patient information sheet which should be given to the patient immediately, so they understand the process. Use this sheet to check off the results when you return to discuss their test results. (Attached to email).

I am closely following the safety and impact of this new process in terms of number of D-dimer tests ordered, number of CTPE scans and number of CTPE scans done without first ordering a D-dimer, or with a normal D-dimer. I am capturing time in ED and whether there are missed cases of PE.

You will receive feedback on your rates for each of the above. The data I see is de-identified, so I will not see your specific feedback.

Questions

Questions

Can I order a CTPE without speaking to the radiologist?

Only using this order set. If you want to order a CTPE without using the order set, you have to call the radiologist.

Why should I change to use the new order set?

Currently we are ordering CTPEs for the wrong patients, and we are ordering unnecessary CTPE scans.

Can I adjust my D-dimer?

You cannot age-adjust your D-dimer threshold using this order set. No D-dimer edits are permitted. You also should not age-adjust your D-dimer unless YOU DOCUMENT that the Wells score for PE is <4. Our data shows that virtually no-one in our group records the Wells score for PE.

Can I use PERC?

Yes, if your normal practice is to use PERC, use it prior to reaching for the PE testing order set and DOCUMENT THE PERC SCORE in your chart.

Why does the order set not use the Wells score for PE?

We showed that the Wells score for PE is frequently wrongly calculated, it adds cognitive burden and takes time to do properly in the ED. We have scrapped the Wells score in favour for stand alone Ddimer.

Can D-dimer rule out PE in all patients? I thought it was only for use in low probability patients.

D-dimer assays have improved greatly over time. Since January 2018, no patient with a negative Ddimer (<500) has been diagnosed with PE. In the YEARS study, the ADJUST-PE study and our recent PEGED study, there was no patient diagnosed with PE who had a negative D-dimer. Yes, you can rule out PE with a negative D-dimer (<500).

Why do I have to check off the YEARS components if we are not using this score?

The YEARS algorithm requires everyone to have a D-dimer. If the D-dimer is <500 then PE is ruled out for everyone.

The YEARS algorithm also allows you to use a higher D-dimer threshold of 1000 of anyone who has none of the following - PE most likely diagnosis / leg swelling and tenderness / hemoptysis. I will analyze whether it would have been safe for us to use this process after collecting 6 months of data.

To do this, I need you to check off whether there are any YEARS components or not.
Why is Kerstin so sure this is a better pathway than how I normally diagnose PE?
Currently too many patients and the wrong patients are having CTPE scans. This new pathway is the culmination of 71 qualitative emergency physician interviews and 18 months building a database to collect information on every patient tested for PE in our emergency department. My MSc student ran additional 30 qualitative interviews with patients who were being tested for PE. I have analyzed the data on every patient who was tested for PE in HHS emergency department since January 2018 and now have a real-time database which continues to capture our data from month to month. Every stakeholder has been involved in developing this pathway including you, patients, radiologists, the hematology lab and the Thrombosis team. This pathway will standardize our practice and the impact will be reported back to all of you.
Further podcast material on this topic is available here
https://emergencymedicinecases.com/tag/dr-kerstin-dewit/
Do not hesitate to contact me with questions or concerns. I favour not responding with 'reply all'. In addition to responding to each of you personally, I will send another email with a summary of answers in a week if I have repeated questions.
Thank for your time. I appreciate that change feels awkward and difficult but I am asking each of you to make a change as of Monday.

Kerstin

Table S1:

CEDIS description	Pts #	% of total CTPAs
**Initially included**		
Shortness of Breath	366	35.3%
Chest Pain (Cardiac Features)	261	25.2%
Chest Pain (Non-Cardiac)	98	9.5%
**Subtotal**	**725**	**69.9%**
**Included after checking 2018 data**		
Heart Palpitations	43	4.1%
Syncope/Pre-Syncope	29	2.8%
Hemoptysis	16	1.5%
Cardiac arrest (non-traumatic)	7	0.7%
Respiratory Arrest	1	0.1%
Syncope/Pre-Syncope	1	0.1%
General Weakness	43	4.1%
Back Pain/Dorsalgia	24	2.3%
Cough/Congestion	18	1.7%
Abdominal Pain Unspecified	25	2.4%
**Subtotal**	**207**	**20.0%**
**Not included**		
Altered level of Consciousness	9	0.9%
Confusion/Disorientation	6	0.6%
Imaging/Blood Test	6	0.6%
Fever	5	0.5%
Lower Extremity Pain	5	0.5%
Abnormal Lab/Imaging	5	0.5%
Localized Swelling	5	0.5%
Vomiting	4	0.4%
Flank Pain	4	0.4%
Hypertension	4	0.4%
Postoperative Complication	4	0.4%
Nausea and/or Vomiting	4	0.4%
Vomiting Blood	3	0.3%
Leg Swelling/Edema	3	0.3%
Upper Extremity Pain	3	0.3%
Head Injury	3	0.3%
Allergic Reaction	2	0.2%
Dizziness/Vertigo	2	0.2%
Minor Complaints NOS	2	0.2%
Lower Extremity Pain	2	0.2%
Neck Swelling/Pain Lo	2	0.2%
Unilateral Reddened H	2	0.2%

Upper Extremity Pain	2	0.2%
General Weakness	2	0.2%
Seizure	1	0.1%
Urinary Retention	1	0.1%
Back Pain/Dosalgia	1	0.1%
Cyanosis	1	0.1%
Edema (Generalized)	1	0.1%
Hematuria	1	0.1%
Abnormal Lab/Imaging	1	0.1%
Constipation	1	0.1%
Extremity Weakness SX	1	0.1%
Flank Pain	1	0.1%
Head Injury	1	0.1%
Medical Device Problems	1	0.1%
Neck Swelling/Pain Lo	1	0.1%
Substance Misuse/Into	1	0.1%
Urinary Tract INF Com	1	0.1%
Wound Check	1	0.1%
**Subtotal**	**105**	**10.1%**

Figure S1: time trend for testing in adherence to the protocol

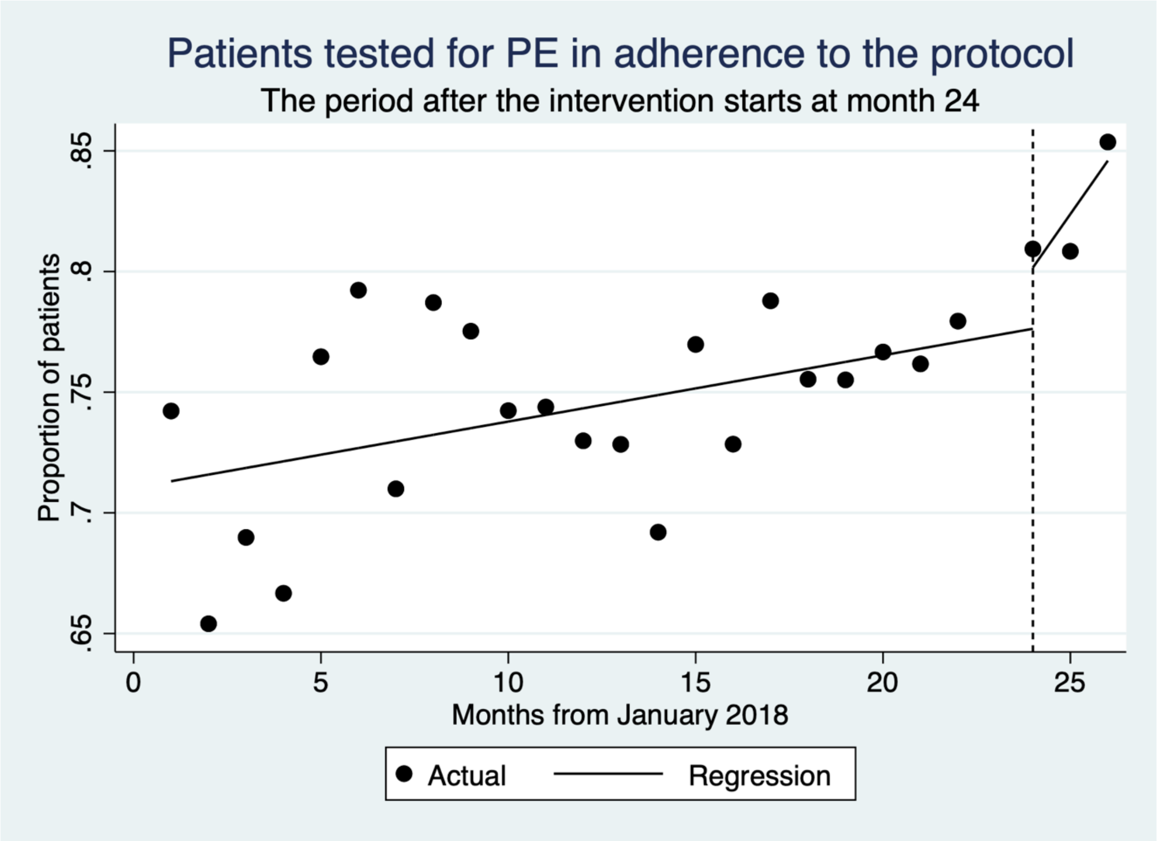

Figure S2: time trend for positive yield of image tests

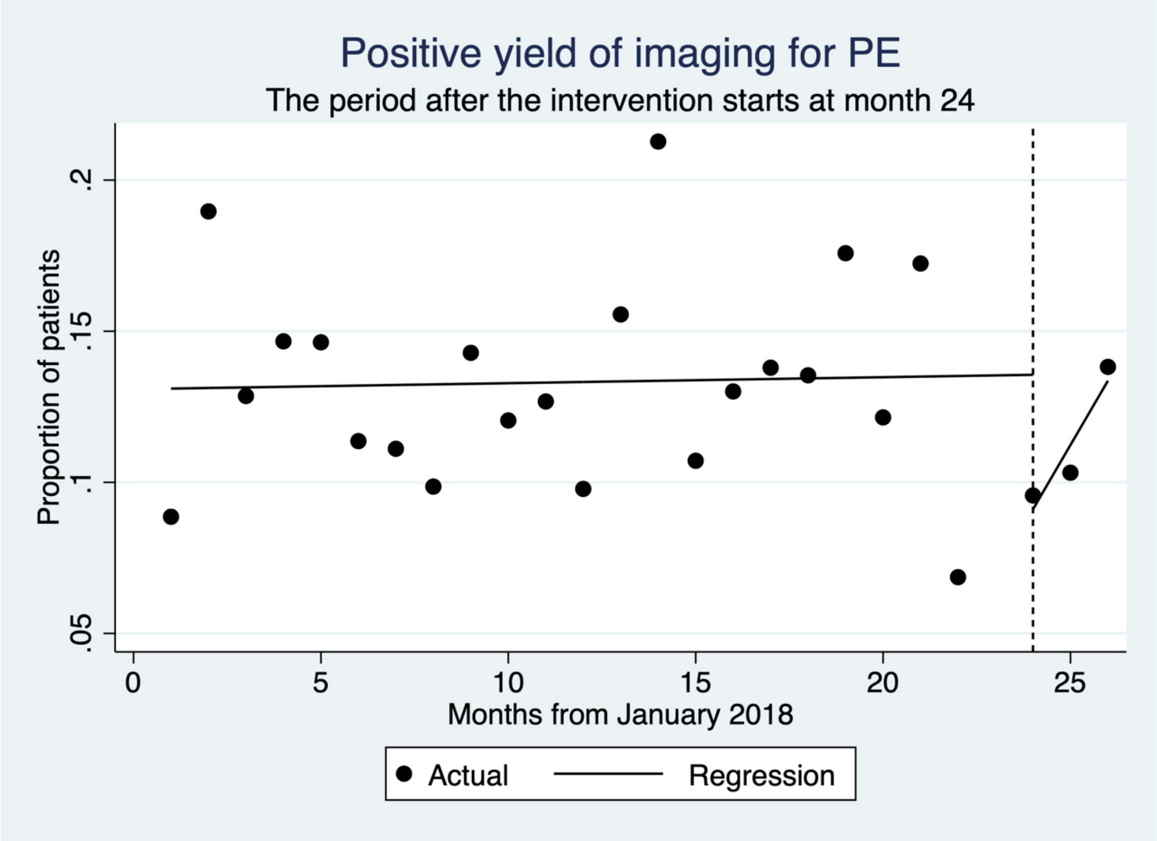

Figure S3: time trend for imaging tests ordered not following the protocol

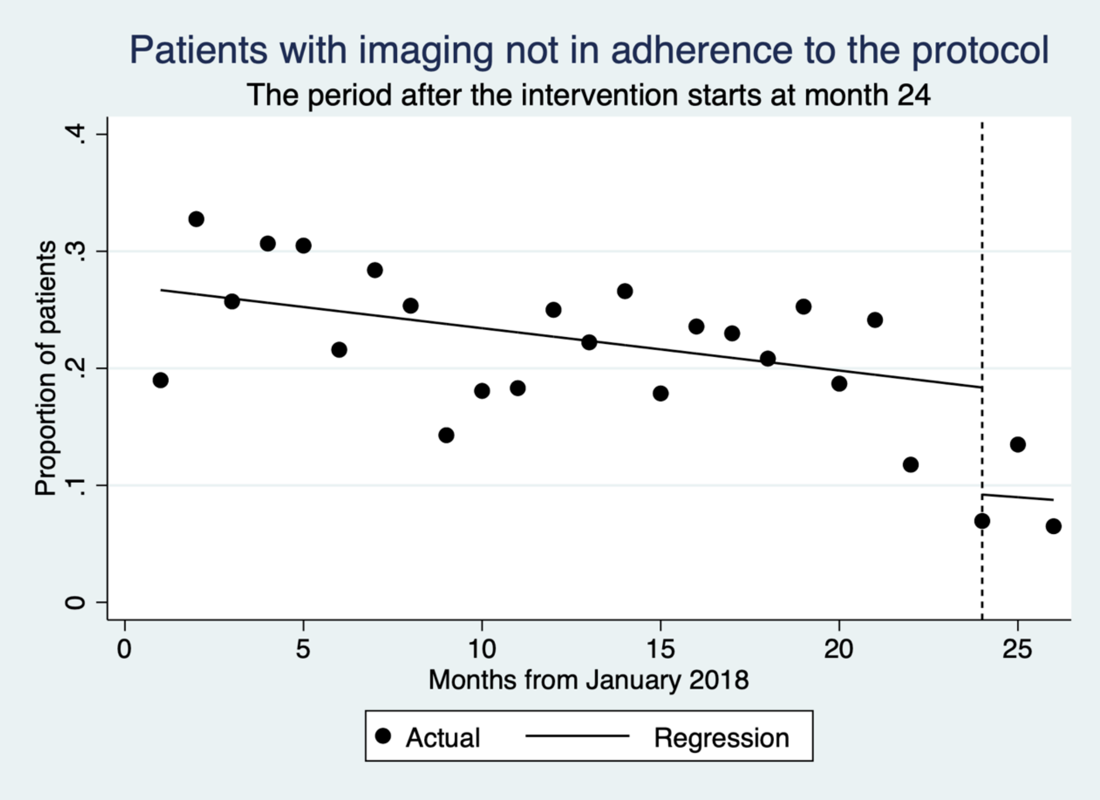

Figure S4: time trend for PE prevalence

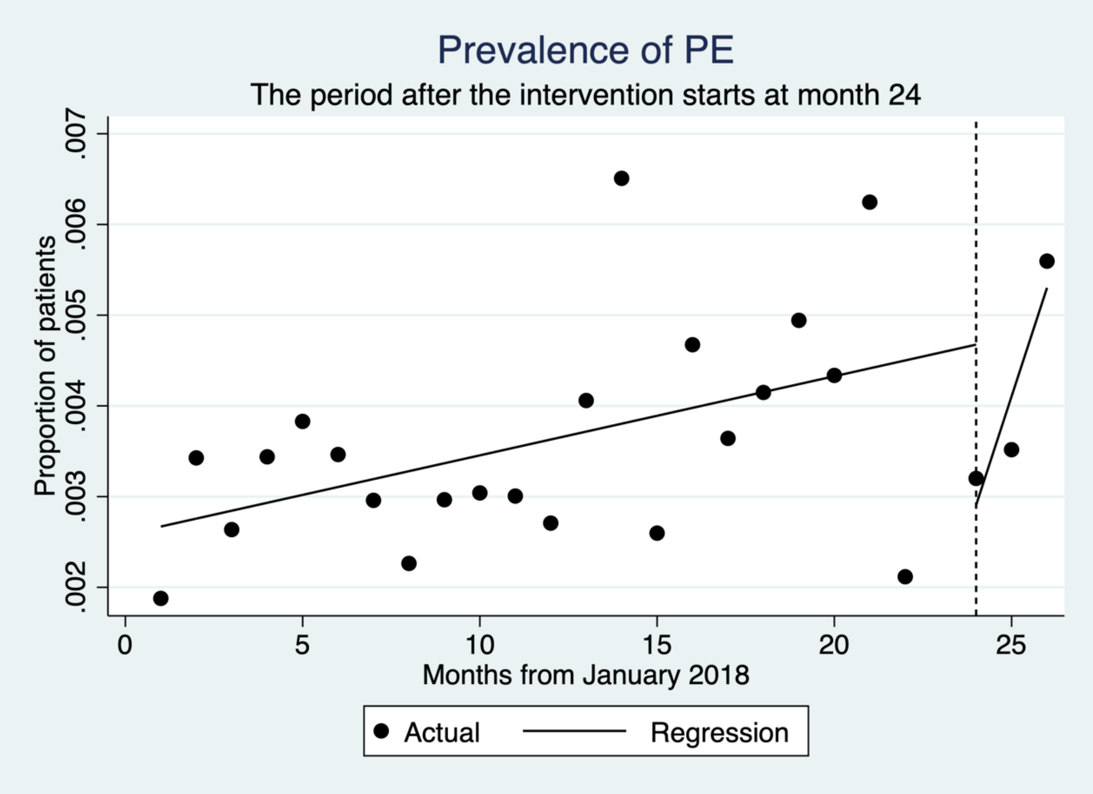

Figure S5: time trend for prevalence of central PE

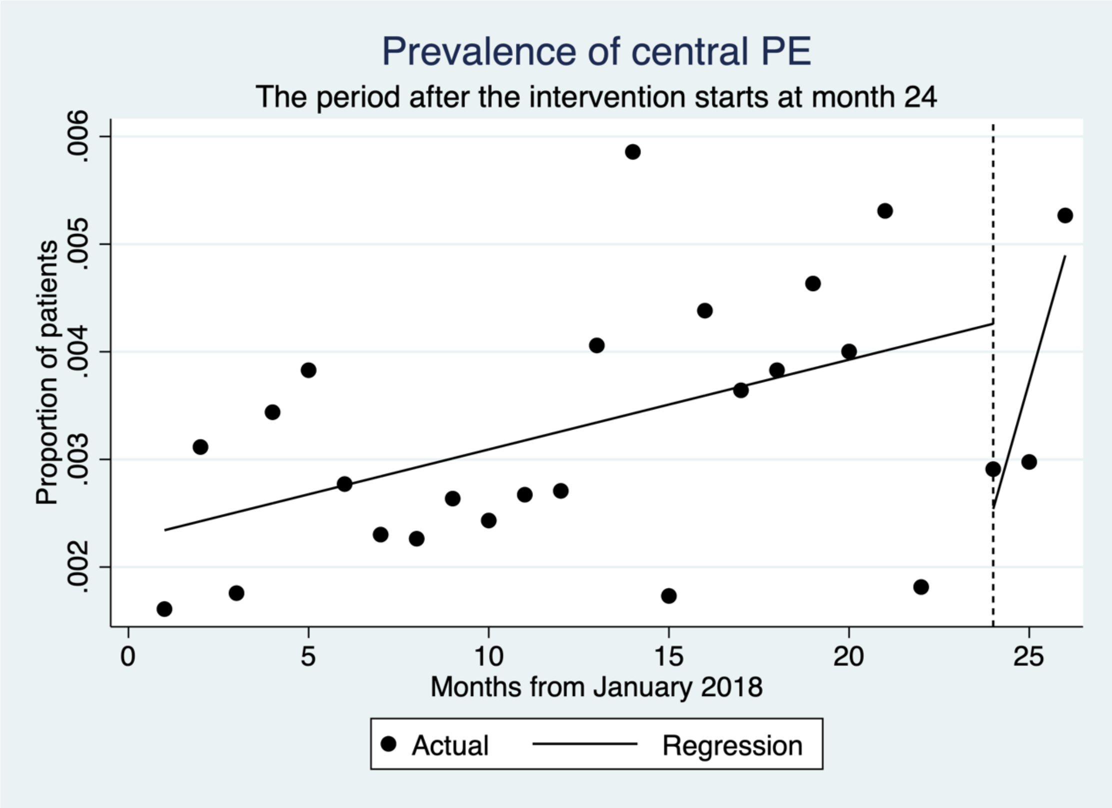

Figure S6: time trend for prevalence of distal PE

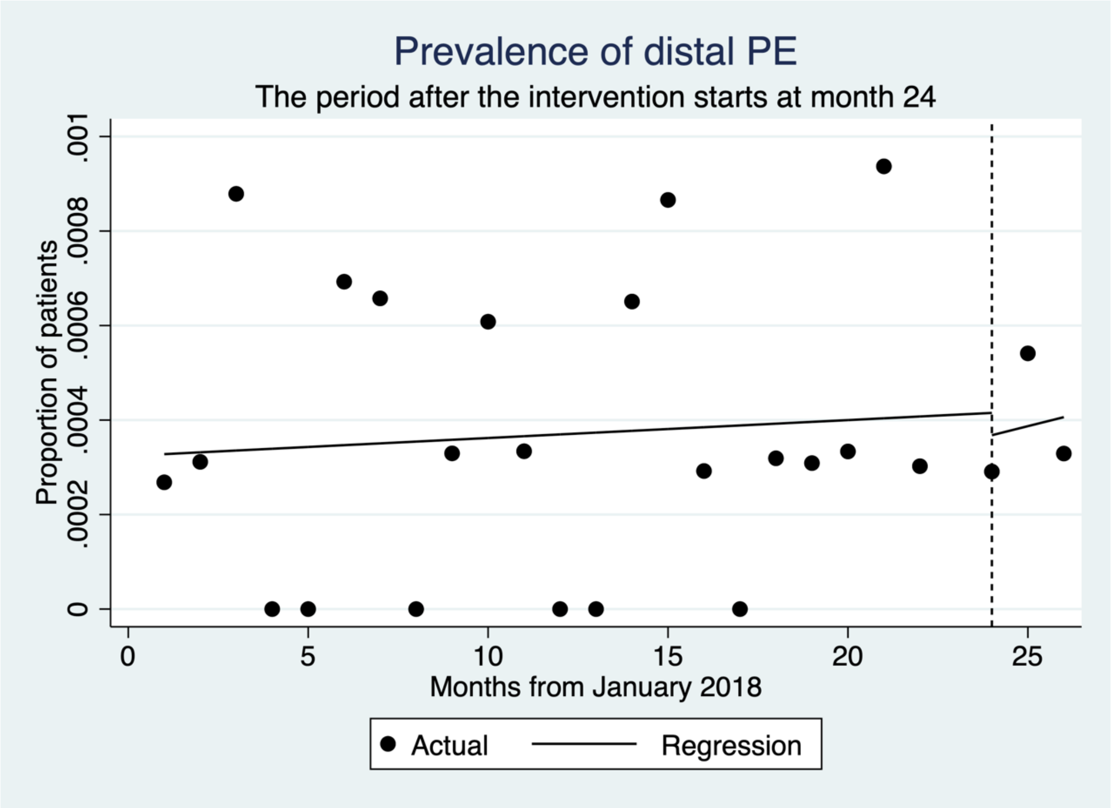

Supplement: Supplementary file 1 — Additional file 1: Appendix A. PE testing order set. Appendix B. Text of the email sent to the ED physician before introducing the order set. Table S1. Figure S1. Time trend for testing in adherence to the protocol. Figure S2. Time trend for positive yield of image tests. Figure S3. Time trend for imaging tests ordered not following the protocol. Figure S4. Time trend for PE prevalence. Figure S5. Time trend for prevalence of central PE. Figure S6. Time trend for prevalence of distal PE. [file 40814_2020_741_MOESM1_ESM.pdf]
